# Supplementary material for: Deep sequencing of pre-translational mRNPs reveals hidden flux through evolutionarily conserved alternative splicing nonsense-mediated decay pathways
Source: Genome Biol. 2021 May 3;22:132. doi: 10.1186/s13059-021-02309-y (PMC8091538; doi:10.1186/s13059-021-02309-y)
Supplement: Supplementary file 4 — Additional file 4. Includes peer review history. [file 13059_2021_2309_MOESM4_ESM.docx]

**1^st^ round**

**Reviewer 1**

This paper by Moore and colleagues extends their use of RIPit-Seq to investigate splicing patterns prior to NMD. The data and clean, convincing and rigorously performed. The conclusion that AS-NMD is more pervasive than previously observed is strongly supported by the data and important for the field. In particular, the data in Supplemental Table 3 will be a useful resource for other researchers. My only minor comment to strengthen the work is that MaxENT is not a great predicter of 3' splice site strength. The fact that the authors observe less robust distinction between error and noise based on 3'ss scores is consistent with this. It would be interesting therefore to compare results using another 3'ss prediction program.

**Reviewer 2**

Alternative splicing generates an enormous diversity of functional and non-functional transcript isoforms in human cells. A major subset of non-functional transcript isoforms is subject to nonsense-mediated decay due to the occurrence of stop codons >50 nt upstream of the last exon junction. Because many NMD targets are efficiently degraded, it can be difficult to fully characterize splice isoform diversity using standard methods. Here, Kovalak and colleagues use RNA-Seq datasets generated with the RIPiT technique, an approach previously developed by the Moore lab to map the locations of exon junction complexes transcriptome-wide, to investigate transcript isoforms enriched in association with the EJC relative to total or cytoplasmic RNA. The authors report enhanced identification of AS-NMD targets and identify splicing events not represented in several major transcript annotation databases. However, the analyses are confounded by a lack of proper control datasets, limiting the utility and significance of the findings.

1. The authors justify their choice of whole-cell and cytoplasmic RNA-Seq datasets on the basis of their "similarity in cell treatment and library preparation" to the RIPiT libraries. However, the conditions used to produce the datasets are different in several important respects, making conclusive comparisons difficult or impossible. The comparison between RIPiT and standard RNAseq is central to the arguments in the paper, so this is a major problem that must be addressed.

a. Contrary to the assertion that the cells were treated similarly, the RIPiT protocol was conducted on cells treated for 1 hour with the translation inhibitor harringtonine, while the total and cytoplasmic RNA datasets were generated from untreated cells. Harringtonine treatment inhibits translation-dependent decay pathways, so the underlying transcriptome will be different in treated and untreated cells, making it impossible to unambiguously attribute increased identification of AS-NMD transcript isoforms to EJC co-purification rather than NMD inhibition. This is a critical difference and calls into question the idea that RIPiT gives a particular advantage for identification of novel splice sites over simply inhibiting translation and/or NMD and sequencing more deeply.

b. The RIPiT libraries were generated from larger RNA fragments and subjected to 150 bp paired-end sequencing vs. 50 bp paired-end sequencing of the total and cytoplasmic RNA libraries. This is a second major source of bias that could lead to better identification of novel splice junctions in the RIPiT data (see for example Genome Biol. 2015; 16(1): 131, which demonstrated vastly better identification of known and novel splice junctions with 100 bp reads vs. 50 bp reads). This analysis is consistent with Figure 3D, which shows that the standard RNA-Seq data identifies annotated junctions much more poorly than the RIPiT.

c. The basis for saying that the library preparation was similar is unclear, since the libraries were prepared using two different approaches (Illumina TruSeq with RiboZero depletion for RNA-Seq and Clontech SMARTer smRNA-Seq Kit for RIPiT).

2. Figure 2: Panels B-E show box and scatter plots of TPM values from EJC and whole cell or cytoplasmic RNA-Seq quantification of different Ensembl transcript annotation classes. The authors use these plots to support the idea that RIPiT enriches for transcripts in the "NMD", "retained intron", "NSD", and "processed transcript" classes. There are typically many Ensembl-annotated transcripts for each gene (leading to numbers such as 39,014 "protein-coding" isoforms in 2C), most of which overlap. This confounds the analysis, in ways that may either over- or under-estimate the extent of the difference between transcript classes. For statistical analyses, it is inappropriate to count multiple transcripts as independent events if the underlying transcript quantifications are based on largely overlapping sequences. This leads to inflated n's and artificially low p values. Conversely, the large degree of overlap between "NMD" isoforms and "protein-coding" isoforms means that the observed change in enrichment of NMD isoforms may be artificially small. It would be much better to analyze individual junctions/splicing events that create "normal" or "NMD" isoforms, rather than using overall transcript abundance.

3. The locations and read counts from all known and novel junctions used for the analysis should be included in the Supplemental information. Also, the Supplemental Table 3 file available for download contains only a list of gene names, which does not match the legend.

4. At multiple points in the manuscript the authors contrast their identification of new junctions in ~60 million RIPiT reads with CHESS annotations derived from 900 billion reads. This is not an appropriate comparison, as the CHESS annotations use several fairly stringent criteria for the inclusion of novel transcript isoforms in the database. The overall diversity of transcripts observed in GTEX data is staggeringly larger than that represented in the CHESS annotations. As noted in the CHESS paper (Genome Biol. 2018; 19: 208), "Although this study greatly increases the number of isoforms of known genes, the 323,258 transcripts reported here represent just 1.1% of the 30,467,424 distinct transcripts observed across all 9795 data sets." Therefore, it is highly likely that the junctions identified here are represented in the 900 billion GTEX reads but fail to meet the CHESS annotation criteria.

5. Figures 1B-D and S1B-D: A major claim of the paper is that RIPiT more accurately measures "flux" through the NMD pathway. This is largely supported by analysis of six individual genes shown in these figures, for which only four are PSI values reported. Of these, one (snRNPA1) has a higher PSI for the poison exon in the cyto/total RNaseq than the RIPiT. Without a more comprehensive analysis of PSI values of poison exons, it remains unclear whether RIPiT has a systematic advantage for capturing flux.

Minor points:

1. Figure 1: Information about how PSI values were calculated should be provided

2. The exon conservation scale from Figure 6B overlaps the graph in 6A.

3. Results paragraph 5: Many RNAs annotated as "lincRNAs" in the Ensembl database have been found to be translated, some of which are also targeted by NMD.

4. While highly likely to be true, the statement "we conclude that the splicing machinery has no ability to read frame" doesn't logically follow from the data presented. It would be reasonable to say that the data show no evidence of reading frame detection by the splicing machinery, but not to rule it out based on the evidence provided. This whole line of argument is quite tangential to the rest of the paper, and I suggest omitting it.

5. Figure 3A: The Gencode annotations are derived from Ensembl annotations, so it's redundant to list them both.

6. Figure 2, Panels B-E: The scatter plots are a poor way to visualize data of this type, especially for comparison among transcript classes. Reporting ratios of EJC TPM:whole cell TPM, etc., for each transcript class on the same graph would be much clearer. Statistical analyses are needed to assess differences among the classes in panels C-E.

**Reviewer 3**

This paper describes an analysis of the population of mRNAs that are associated with exon junction complexes, which represent RNAs that have undergone splicing but have generally not yet been translated. This population of RNAs is of interest as it represents the initial products of pre-mRNA splicing, prior to the action of various quality control mechanisms (such as nonsense-mediated mRNA decay, NMD) that degrade specific mRNAs in the cytoplasm, mechanisms that generally require translation. The approach is based on deep sequencing libraries prepared by the lab's previously described EJC RIPiT approach, followed by computational analysis. The data appear to be of high quality, and produce expected results in genes which are known to undergo extensive NMD of alternatively spliced isoforms (Fig. 1), and also pass various other sanity checks (Fig. 2). These data detect thousands of unannotated splice junctions representing various types of alternative splicing as well as the expected annotated junctions, and evidence is presented that the differences between RIPiT sequencing and RNA-seq can largely be attributed to NMD. This is not a big surprise, but is an important confirmatory result. There is also some analysis of splice site properties, evolutionary conservation and so forth, that flesh out the picture of these transcripts. Overall, the characterization of this population of RNAs is important as it has not been previously characterized in a genome-wide fashion. The analyses and comparisons to available annotation are carefully done and generally well described. Attention to three main issues would significantly strengthen the paper:

1. The EJC RIPiT sequencing data are not terribly deep, just 3 replicates of 18-25 million read pairs each, yielding 6-10 M aligned RIPiT read pairs per replicate, compared to 30-43 M read pairs per replicate in the RNA-seq datasets used. For a paper that addresses alternative mRNA isoforms in a genome-wide fashion in the year 2019, this depth seems quite limited. How many more unannotated junctions, especially in lower-expressed genes, would be detected if the sequencing were, say, 3X deeper, so as to approach the depth of mapped read pairs in the RNA-seq data?

2. The authors note that they exposed cells to the translational inhibitor harringtonine for 60 minutes to enrich for pre-translational mRNAs, which might trigger regulatory responses in the cells, and that "Because any transcriptional effects would confound the analysis, elimination of translation inhibitors would be advisable for any future EJC RIPiT-Seq study specifically aimed at quantifying flux through alternative RNA processing pathways in non-perturbed cells." Since quantifying this flux was one of the goals of this study, this begs the question of why the authors didn't perform the RIPiT analysis without translational inhibitors to assess whether significant transcriptional compensation may have occurred.

3. The paper is quite descriptive in many parts, with very few statistical analyses presented to bolster the authors' claims. Statistical significance is assessed in Figures 2B and 4B, but virtually nowhere else in the manuscript. The differences between RIPiT and RNA-seq data in Figure 1 are very large, so statistics are perhaps not essential, but various results shown in Figures 3, 5 and 6 need to be assessed by appropriate statistical tests.

Minor points:

1. The fraction of unannotated junctions that result from splicing by the minor spliceosome is underestimated. The authors note that just 3 of 5,412 unannnotated junctions have AT-AC termini and conclude that the fraction is under 0.1%, but only about ¼ to 1/3 of minor spliceosomal introns have AT-AC termini (e.g., Levine and Durbin NAR 2001), so it would be more accurate to multiply this number by 3 or 4, yielding an estimate closer to 0.2%, roughly matching the estimated proportion of minor class introns in the human genome.

2. The discussion of flux through AS-NMD pathways should probably also cite Lewis et al. 2003 (PMID 12502788), which I believe was the first paper to demonstrate that a large number of genes use the AS-NMD pathway.

**Authors’ response**

We thank all three reviewers for their time and expertise in reviewing this manuscript. We are grateful for their detailed comments and insightful suggestions, which we carefully considered. These suggestions enabled us to make numerous improvements to the manuscript, including significant additional data and multiple revisions to clarify the figures and text.

Major changes:

- We constructed new RNA-Seq libraries whose insert sizes better matched the EJC RIPiT-Seq libraries. These new libraries have 3 biological replicates each from HEK293 cells subjected (+) or not (-) to a one hour pre-treatment with harringtonine. Like the EJC libraries, the new RNA-Seq libraries were sequenced as paired-end, 150 bp reads.
- All figures and tables have been updated to include the new RNA-Seq data, and the old RNA-Seq libraries [previous reference 14] have been removed.
- Because we no longer include whole cell and cytoplasmic RNA-Seq libraries, we remade Figure 1A to illustrate the expected differences between EJC and whole cell RNA-Seq libraries from cells subjected (+) or not (-) to harringtonine pre-treatment.
- The previous Figure 2D is now Supplemental Figure 2A. This figure now includes boxplots (like those in Figure 2B) to show the TPM distribution on unspliced and spliced transcripts in all library types.
- Figure 2E and Supplemental Figure panels 2A-C have been removed.
- We calculated transcript abundance ratios between the three library types. This analysis is now included as the new Supplemental Figure 2B.
- We calculated read counts spanning individual junctions in addition to our analysis of overall transcript abundance. Figure 2D now shows the distribution of read counts at unique junctions (i.e., splicing events only found in a single transcript isoform) in our libraries. The new Figure 2E shows read count ratios at these junctions between the three library types.
- As a result of updating Figure 4A with the new RNA-Seq libraries, we have added an analysis of position +5 to Figure 4B. This figure now also includes sequence motifs of the splice sites used at positions +3, +4, and +5.
- We have added statistical analysis of Figures 5B and C as our new Supplemental Figure 4C.
- To further support our conclusion that the one hour harringtonine treatment had a minimal effect on transcript expression, we analyzed previously published RNA-Seq libraries made from HeLa cells treated with cycloheximide for 0 minutes, 15 minutes, or 24 hours [36]. Transcriptome-wide expression differences among these libraries is now included in the new Supplemental Figure 6A. In the new Supplemental Figure 6B, we look specifically at the AS-NMD isoforms highlighted in Figures 1B-D and Supplemental Figures 1B-D.

Minor changes:

- The PSI and PSO values in Figures 1B-D, 5D-E, 6C-D, and Supplemental Figures 1B and D, 5A-C were calculated using the formulae now provided in the Methods section and have been updated.
- Supplemental Figure 1A now includes more scatter plots comparing the additional RNA-Seq replicates.
- Supplemental Figure 1C has been changed to a percent spliced out (PSO) calculation to more clearly show the frequency of the exon-skipping NMD event, and this formula is also now provided in our Methods section.
- The PIR values in Supplemental Figure 4C were calculated using the formula that is now provided in the Methods section and have been updated.
- The isoform counts in Figure 2B now reflect the total number of expressed isoforms in our datasets rather than the number of annotated isoforms.
- We have added a statistical analysis of Figure 3D in the text.
- We have added a statistical analysis of Figure 6A in the figure.

Our detailed responses to individual reviewers’ comments are shown below in blue.

---------------------------------------

## Reviewer #1: This paper by Moore and colleagues extends their use of RIPit-Seq to investigate splicing patterns prior to NMD. The data and clean, convincing and rigorously performed. The conclusion that AS-NMD is more pervasive than previously observed is strongly supported by the data and important for the field. In particular, the data in Supplemental Table 3 will be a useful resource for other researchers.

Thank you. We agree! Supplemental Table 3 has now been updated to reflect the new RNA-Seq data plus junction read counts for all libraries.

My only minor comment to strengthen the work is that MaxENT is not a great predicter of 3' splice site strength. The fact that the authors observe less robust distinction between error and noise based on 3'ss scores is consistent with this. It would be interesting therefore to compare results using another 3'ss prediction program.

Based on this comment, we tried to analyze our data using multiple other 3’ splice site strength algorithms. Unfortunately, we hit roadblocks at every turn:

- **NatGene2** (http://www.cbs.dtu.dk/services/NetGene2/instruct.php): Analyzes much larger (>200 nt) regions than MaxEntScan (23 nt); NatGene2 then predicts the best splice sites in that region, rather than scoring user-defined splice sites.
- **Splice Site Score Calculation** (http://rulai.cshl.edu/new_alt_exon_db2/HTML/score.html): Allows for only individual splice site entry – unfeasible for a dataset of our magnitude
- **Analyzer Splice Tool** (http://ibis.tau.ac.il/ssat/SpliceSiteFrame.htm): Website no longer functional
- **Human Splicing Finder** (http://www.umd.be/HSF/): Allows for only individual unannotated splice site entry – unfeasible for a dataset of our magnitude
- **GeneSplicer** (http://ccb.jhu.edu/software/genesplicer/): Website no longer functional
- **NNSplice** (https://www.fruitfly.org/seq_tools/splice.html): Must rely on NNSplice to identify the splice sites, many of our unannotated splice sites could not be identified this way

In their 2015 paper, Jian, Boerwinkle and Liu (PMID: 24263461) provided a detailed survey of in silico tools for splice site prediction from the user’s viewpoint. Quoting from that article:

*“To date the most unbiased approximation for modeling short sequence motifs is to use the Maximum Entropy Distribution (MED). Compared with other methods, the only assumption of MED is the consistency with the features of the empirical distribution estimated from available data. MED also considers dependencies between both non-adjacent and adjacent positions. Rather than a single model, MED is a framework with much flexibility for generating different models by simply changing the sets of constraints. The approach has been utilized by the tool MaxEntScan[.]”*

Given the difficulties we encountered trying to use other tools, and that this comparison was only a “minor” suggestion by this reviewer, we have chosen not to pursue this avenue any further.

---------------------

## Reviewer #2: Alternative splicing generates an enormous diversity of functional and non-functional transcript isoforms in human cells. A major subset of non-functional transcript isoforms is subject to nonsense-mediated decay due to the occurrence of stop codons >50 nt upstream of the last exon junction. Because many NMD targets are efficiently degraded, it can be difficult to fully characterize splice isoform diversity using standard methods. Here, Kovalak and colleagues use RNA-Seq datasets generated with the RIPiT technique, an approach previously developed by the Moore lab to map the locations of exon junction complexes transcriptome-wide, to investigate transcript isoforms enriched in association with the EJC relative to total or cytoplasmic RNA. The authors report enhanced identification of AS-NMD targets and identify splicing events not represented in several major transcript annotation databases. However, the analyses are confounded by a lack of proper control datasets, limiting the utility and significance of the findings.

1. The authors justify their choice of whole-cell and cytoplasmic RNA-Seq datasets on the basis of their "similarity in cell treatment and library preparation" to the RIPiT libraries. However, the conditions used to produce the datasets are different in several important respects, making conclusive comparisons difficult or impossible. The comparison between RIPiT and standard RNAseq is central to the arguments in the paper, so this is a major problem that must be addressed.

We agree that this was a significant weakness in our original submission. Therefore, we now compare our EJC RIPiT-Seq libraries to newly created HEK293 whole cell RNA-Seq libraries (three biological replicates) wherein the captured fragments are of similar length (220-500 nts) to our previously published EJC RIPiT-Seq libraries. While the absolute numbers of read counts and splice junctions detected have changed in multiple places (as noted in the text), the only major conceptual changes from this more appropriate comparison are:

**Figure 3D**: RPM supporting annotated junctions are now nearly identical between the EJC and RNA-Seq libraries. But reads supporting unannotated junctions are still higher in the EJC libraries. We have changed both the figure and text describing it to reflect this. In the Discussion, we have also removed the claim that EJC RIPiT-Seq is better at detecting low abundance spliced isoforms not subject to rapid translation-dependent decay.

**Figure 6A**: There are now more splice junction reads supporting our newly-identified cassette exons in the RNA-Seq datasets. Nevertheless, the differences in splice junction read counts on cassette exons detected in both EJC and RNA-Seq libraries remain statistically significant, with the EJC libraries still returning substantially more reads per million than the RNA-Seq datasets. Of particular note was our finding that the number of novel cassette exons solely detectable in EJC libraries (now N = 318) is almost the same as in our original report (N = 315).

a. Contrary to the assertion that the cells were treated similarly, the RIPiT protocol was conducted on cells treated for 1 hour with the translation inhibitor harringtonine, while the total and cytoplasmic RNA datasets were generated from untreated cells. Harringtonine treatment inhibits translation-dependent decay pathways, so the underlying transcriptome will be different in treated and untreated cells, making it impossible to unambiguously attribute increased identification of AS-NMD transcript isoforms to EJC co-purification rather than NMD inhibition. This is a critical difference and calls into question the idea that RIPiT gives a particular advantage for identification of novel splice sites over simply inhibiting translation and/or NMD and sequencing more deeply.

We agree that this was another significant weakness of our original manuscript. To address this, we generated our new RNA-Seq libraries from cells that were (+) or were not (-) subjected to a one hour pre-treatment with harringtonine. These new libraries were also sequenced more deeply (84-93 million mate pairs each) than the RNA-Seq libraries analyzed in the original manuscript (51-57 million mate pairs each).

Importantly, comparison of the harringtonine (+) and (-) RNA-Seq libraries now clearly demonstrates that one hour inhibition of translation in non-confluent HEK293 cells results in no detectable increase in the abundance of AS-NMD isoforms (**Figure 1B-D** and **Supplemental Figure 1B-D**). Thus our conclusion that EJC RIPiT-Seq is advantageous for identifying AS-NMD isoforms and assessing flux through protein-coding versus AS-NMD pathways holds.

As an additional comparison, we’ve also now analyzed whole cell RNA-Seq datasets from HeLa cells subjected to no, 15 min or 24 hour pretreatment with cycloheximide, another translation inhibitor (Kearse ... Wilusz, 2019, Genes Dev; PMID: 31171704). In these libraries, both the 15 min and 24 hour cycloheximide pretreatments did lead to detectable increases AS-NMD isoform abundance for our known AS-NMD targets, with the 24 hour pretreatment having a larger effect than the 15 min pretreatment (new **Supplemental Figure 6B**). The 24 hour pretreatment, however, did lead to changes in the overall transcriptome (see Supplemental Figure 7A in Kearse … Wilusz, 2019, and our new **Supplemental Figure 6A**). Thus while prolonged translation inhibition may present a viable alternative to purification of pre-translational RNPs for the identification of new AS-NMD isoforms, any interpretation must consider possible pleiotropic effects due to prolonged translation inhibition. These points have now been added to the Discussion section.

b. The RIPiT libraries were generated from larger RNA fragments and subjected to 150 bp paired-end sequencing vs. 50 bp paired-end sequencing of the total and cytoplasmic RNA libraries. This is a second major source of bias that could lead to better identification of novel splice junctions in the RIPiT data (see for example Genome Biol. 2015; 16(1): 131, which demonstrated vastly better identification of known and novel splice junctions with 100 bp reads vs. 50 bp reads). This analysis is consistent with Figure 3D, which shows that the standard RNA-Seq data identifies annotated junctions much more poorly than the RIPiT.

The reviewer is correct. As discussed above, this problem has now been rectified with our new RNA-Seq libraries. Please see our point above about revised **Figure 3D**.

c. The basis for saying that the library preparation was similar is unclear, since the libraries were prepared using two different approaches (Illumina TruSeq with RiboZero depletion for RNA-Seq and Clontech SMARTer smRNA-Seq Kit for RIPiT).

We have now removed this claim.

2. Figure 2: Panels B-E show box and scatter plots of TPM values from EJC and whole cell or cytoplasmic RNA-Seq quantification of different Ensembl transcript annotation classes. The authors use these plots to support the idea that RIPiT enriches for transcripts in the "NMD", "retained intron", "NSD", and "processed transcript" classes. There are typically many Ensembl-annotated transcripts for each gene (leading to numbers such as 39,014 "protein-coding" isoforms in 2C), most of which overlap. This confounds the analysis, in ways that may either over- or under-estimate the extent of the difference between transcript classes. For statistical analyses, it is inappropriate to count multiple transcripts as independent events if the underlying transcript quantifications are based on largely overlapping sequences. This leads to inflated n's and artificially low p values. Conversely, the large degree of overlap between "NMD" isoforms and "protein-coding" isoforms means that the observed change in enrichment of NMD isoforms may be artificially small. It would be much better to analyze individual junctions/splicing events that create "normal" or "NMD" isoforms, rather than using overall transcript abundance.

We thank the reviewer for both educating us about this problem and suggesting its solution. In response, we have now performed the suggested analysis using only those exon junctions (or intron-exon boundaries for retained introns) that are unique to individual GRCh38.p12 isoforms. As predicted by the reviewer, this revealed even higher enrichment of NMD, NSD, retained intron and processed transcripts in EJC than RNA-Seq libraries. These data are now presented as **new Figure 2D and E**.

3. The locations and read counts from all known and novel junctions used for the analysis should be included in the Supplemental information. Also, the Supplemental Table 3 file available for download contains only a list of gene names, which does not match the legend.

We have now modified **Supplemental Table 2** to include the requested information for annotated junctions (i.e., read counts in every biological replicate). Similarly, **Supplemental Table 3** has been modified to include read counts on all unannotated junctions, as well as their genomic coordinates. Although all of the genomic coordinate information was previously contained in Supplemental Table 3, some inadvertent loss of information must have occurred during our initial data upload to the journal site.

4. At multiple points in the manuscript the authors contrast their identification of new junctions in ~60 million RIPiT reads with CHESS annotations derived from 900 billion reads. This is not an appropriate comparison, as the CHESS annotations use several fairly stringent criteria for the inclusion of novel transcript isoforms in the database. The overall diversity of transcripts observed in GTEX data is staggeringly larger than that represented in the CHESS annotations. As noted in the CHESS paper (Genome Biol. 2018; 19: 208), "Although this study greatly increases the number of isoforms of known genes, the 323,258 transcripts reported here represent just 1.1% of the 30,467,424 distinct transcripts observed across all 9795 data sets." Therefore, it is highly likely that the junctions identified here are represented in the 900 billion GTEX reads but fail to meet the CHESS annotation criteria.

Fair point. In the revised manuscript, we have substantially toned down comparisons between our EJC RIPiT-Seq data and CHESS. In the Discussion, we now simply say that “we identified thousands of exon junctions not currently annotated in RefSeq, Ensembl/GENCODE or CHESS”.

5. Figures 1B-D and S1B-D: A major claim of the paper is that RIPiT more accurately measures "flux" through the NMD pathway. This is largely supported by analysis of six individual genes shown in these figures, for which only four are PSI values reported. Of these, one (snRNPA1) has a higher PSI for the poison exon in the cyto/total RNaseq than the RIPiT. Without a more comprehensive analysis of PSI values of poison exons, it remains unclear whether RIPiT has a systematic advantage for capturing flux.

In the case of SNRPA1, there is no poison exon. Rather, the exon skipped isoform causes a frameshift, resulting in a premature stop codon in exon 7. In rereading the original manuscript, we realize that we did not correctly indicate this in the legend to **Figure 1**. The relevant number for the SNRPA1 is “percent spliced out” or PSO for exon 6, which we now show in panel C and explain in the figure legend. The equation used for PSO is given in Methods. We apologize for the previous confusion.

### Minor points:

1. Figure 1: Information about how PSI values were calculated should be provided

Our apologies for this previous omission. We have now provided the equations used for PSI, PSO and PIR calculations in the Methods section.

2. The exon conservation scale from Figure 6B overlaps the graph in 6A.

We have now added additional space between **Figures 6A and 6B** to correct this problem.

3. Results paragraph 5: Many RNAs annotated as "lincRNAs" in the Ensembl database have been found to be translated, some of which are also targeted by NMD.

In reconsidering the content of the paper, it seemed to us that the lincRNA data were a distraction from our main points. Therefore, we have elected to remove this paragraph and all lincRNA figure panels (former **Figure 2E and Supplemental Figure 2C**).

4. While highly likely to be true, the statement "we conclude that the splicing machinery has no ability to read frame" doesn't logically follow from the data presented. It would be reasonable to say that the data show no evidence of reading frame detection by the splicing machinery, but not to rule it out based on the evidence provided. This whole line of argument is quite tangential to the rest of the paper, and I suggest omitting it.

We respectfully disagree with the reviewer on this point. The idea that the splicing machinery can detect reading frame has been stubbornly clung to by Ruth Sperling’s lab for many years (references 24 and 25 in the text, with citations as recent as 2019), and non-cognoscente are still occasionally led astray by her “Suppression of Splicing (SOS)” model. Given that our data directly and definitively speak to this issue, we think it an important point to make. Notably, the new RNA-Seq data (which yielded more counts on more junctions) are even more definitive than before. We have now added a sentence to the abstract calling attention to these results.

5. Figure 3A: The Gencode annotations are derived from Ensembl annotations, so it's redundant to list them both.

The GENCODE FAQ website (<https://www.gencodegenes.org/pages/faq.html>) explains that:

*“The GENCODE annotation is made by merging the manual gene annotation produced by the Ensembl-Havana team and the Ensembl-genebuild automated gene annotation. [...] The gene annotation is the same in both files. The only exception is that the genes which are common to the human chromosome X and Y PAR regions can be found twice in the GENCODE GTF, while they are shown only for chromosome X in the Ensembl file. In addition, the GENCODE GTF contains a number of attributes not present in the Ensembl GTF, including annotation remarks, APPRIS tags and other tags highlighting transcripts experimentally validated by the GENCODE project or 3-way-consensus pseudogenes (predicted by Havana, Yale and UCSC).”*

From reading this, one would naturally assume that the Ensembl and GENCODE annotations are virtually identical. Our own analysis, however, revealed 123 junctions present in Ensembl, but not in GENCODE, and 4,554 junctions present in GENCODE but not in Ensembl (see **Supplemental Table 2**). Therefore, for the sake of completeness, we have chosen to include both Ensemble and GENCODE in Figure 3A and Supplemental Table 2.

6. Figure 2, Panels B-E: The scatter plots are a poor way to visualize data of this type, especially for comparison among transcript classes. Reporting ratios of EJC TPM:whole cell TPM, etc., for each transcript class on the same graph would be much clearer. Statistical analyses are needed to assess differences among the classes in panels C-E.

Here, we disagree with the reviewer regarding scatter plots. In the senior author’s strong opinion, presenting results as only ratios is too often used to hide inconsistencies in the underlying data. We, therefore, like to include scatter plots because they enable the reader to get a sense of the data in its rawest form prior to any mathematical manipulation. Nonetheless, we do agree that ratios have their utility. Thus we now include ratio plots in new **Figure 2E** and new **Supplementary Figure 2B**. We have also performed the requested statistical analyses.

---------------------

## Reviewer #3: This paper describes an analysis of the population of mRNAs that are associated with exon junction complexes, which represent RNAs that have undergone splicing but have generally not yet been translated. This population of RNAs is of interest as it represents the initial products of pre-mRNA splicing, prior to the action of various quality control mechanisms (such as nonsense-mediated mRNA decay, NMD) that degrade specific mRNAs in the cytoplasm, mechanisms that generally require translation. The approach is based on deep sequencing libraries prepared by the lab's previously described EJC RIPiT approach, followed by computational analysis. The data appear to be of high quality, and produce expected results in genes which are known to undergo extensive NMD of alternatively spliced isoforms (Fig. 1), and also pass various other sanity checks (Fig. 2). These data detect thousands of unannotated splice junctions representing various types of alternative splicing as well as the expected annotated junctions, and evidence is presented that the differences between RIPiT sequencing and RNA-seq can largely be attributed to NMD. This is not a big surprise, but is an important confirmatory result. There is also some analysis of splice site properties, evolutionary conservation and so forth, that flesh out the picture of these transcripts. Overall, the characterization of this population of RNAs is important as it has not been previously characterized in a genome-wide fashion. The analyses and comparisons to available annotation are carefully done and generally well described. Attention to three main issues would significantly strengthen the paper:

1. The EJC RIPiT sequencing data are not terribly deep, just 3 replicates of 18-25 million read pairs each, yielding 6-10 M aligned RIPiT read pairs per replicate, compared to 30-43 M read pairs per replicate in the RNA-seq datasets used. For a paper that addresses alternative mRNA isoforms in a genome-wide fashion in the year 2019, this depth seems quite limited. How many more unannotated junctions, especially in lower-expressed genes, would be detected if the sequencing were, say, 3X deeper, so as to approach the depth of mapped read pairs in the RNA-seq data?

We agree that deeper sequencing of the EJC RIPiT-Seq libraries would be highly desirable. However, as the Moore lab at UMass Medical School is now closed, the original EJC RIPiT-Seq samples are no longer readily accessible. Although we were able to co-opt resources at Moderna (Dr. Moore’s current employer) to generate the new RNA-Seq libraries, performing EJC RIPiT-Seq experiments does not align with Moderna’s business mandate. Therefore, our only option is to work with the existing EJC RIPiT-Seq sequencing data.

2. The authors note that they exposed cells to the translational inhibitor harringtonine for 60 minutes to enrich for pre-translational mRNAs, which might trigger regulatory responses in the cells, and that "Because any transcriptional effects would confound the analysis, elimination of translation inhibitors would be advisable for any future EJC RIPiT-Seq study specifically aimed at quantifying flux through alternative RNA processing pathways in non-perturbed cells." Since quantifying this flux was one of the goals of this study, this begs the question of why the authors didn't perform the RIPiT analysis without translational inhibitors to assess whether significant transcriptional compensation may have occurred.

We have now addressed this concern with our new RNA-Seq libraries (see our response to Reviewer 2, comment 1a above). We have also removed the quoted sentence from the revised Discussion.

3. The paper is quite descriptive in many parts, with very few statistical analyses presented to bolster the authors' claims. Statistical significance is assessed in Figures 2B and 4B, but virtually nowhere else in the manuscript. The differences between RIPiT and RNA-seq data in Figure 1 are very large, so statistics are perhaps not essential, but various results shown in Figures 3, 5 and 6 need to be assessed by appropriate statistical tests.

We have now added the requested statistical tests:

- **Supplemental Figure 2A** (previously Figure 2D): Added one-way ANOVA and Tukey’s *post hoc* significance tests
- New **Supplemental Figure 2B**: One-way ANOVA and Tukey’s *post hoc* significance tests
- **Figure 3D** (in the text): Kolmogorov–Smirnov test
- New **Supplemental** **Figure 4C**: Mean differences (with standard errors), 95% confidence intervals and p-values from independent (unpaired) t-tests comparing splice site MaxENT and conservation scores shown in **Figures 5B and C**
- **Figure 6A**: Added one-way ANOVA and Tukey’s *post hoc* significance tests

Minor points:

1. The fraction of unannotated junctions that result from splicing by the minor spliceosome is underestimated. The authors note that just 3 of 5,412 unannnotated junctions have AT-AC termini and conclude that the fraction is under 0.1%, but only about ¼ to 1/3 of minor spliceosomal introns have AT-AC termini (e.g., Levine and Durbin NAR 2001), so it would be more accurate to multiply this number by 3 or 4, yielding an estimate closer to 0.2%, roughly matching the estimated proportion of minor class introns in the human genome.

Thank you for pointing this out. We have now removed this statement from the manuscript.

2. The discussion of flux through AS-NMD pathways should probably also cite Lewis et al. 2003 (PMID 12502788), which I believe was the first paper to demonstrate that a large number of genes use the AS-NMD pathway.

We completely agree that this was a foundational paper of the AS-NMD field, and as such, we cited it as reference [7] in the Background section of the original manuscript in the sentence:

*“This protein homeostasis function is mediated by AS linked to NMD (AS-NMD), wherein the flux through alternate splicing pathways that result in protein-coding and NMD isoforms is subject to tight control [7].”*

However, we had previously failed to cite this paper again in the Discussion. To rectify this unintended omission, we have now added reference [7] as supporting the following sentence that opens the “*Measuring flux through AS-NMD pathways*” section.

*“Since its initial description [7, 30, 31], AS-NMD has increasingly emerged as a key post-transcriptional regulatory mechanism [32-34].”*

**2^nd^ round**

**Reviewer 2**

The revised manuscript by Moore and colleagues is improved in many respects. With some exceptions listed below, the conclusions drawn are generally better justified by the data provided. Despite the improvement, several important issues persist. Please see below for specific comments:

1. I share reviewer #3's concern about the sequencing depth of the RIPiT libraries (6-10 million aligned read pairs per replicate). From Figure 3D and Supplemental Tables 2 and 3, it appears that the majority of the unannotated junctions are represented by only a handful of reads. It's not clear how the reads per million calculations were performed, but it is strange that many junctions have identical RPM counts, with each group of identical RPM counts differing by exactly 0.02558 (see the the stair-step pattern of the EJC curves on the CDF plot in Figure 3D and Supplemental Tables 2 and 3). This suggests that they are not simply the mean of the RPMs from the individual biological replicates, which would be the standard way to represent the data. Are the RPMs listed for EJC and total RNAseq calculated in the same way (the RNAseq data do not show this pattern of repeated RPM values), and how exactly were they calculated? This all raises the further question of how many of the listed "novel" junctions were represented in multiple RIPiT libraries. About half of the listed events have a RPM of less than 0.25, which across 20 million mapped reads in three libraries (depending on how the RPM calculations were done; based on the step-wise nature of the RPMs, I'm guessing an aggregate of all reads across the three replicates was used as the denominator), would only mean 5 reads per junction in total. Also, the response to my previous query says that "read counts in every biological replicate" are provided in Supplemental Table 2 and 3, but there is only one RPM column per condition listed. Junction mapping read counts (along with normalized RPMs) should be provided for all replicates.

2. The discussion of the comparison between the novel junctions identified in this study and annotations has improved, but it still conflates detection of junctions with annotation of junctions. The first sentence of the section "EJC libraries capture new exon junctions" says, "We next wondered whether EJC libraries might contain new transcript isoforms that had previously eluded detection due to their low abundance in RNA-seq," but this remains an incorrect representation of the relationship between their data and the annotation compendia used. As previously stated, it is highly likely that most or all of these junctions have been "detected," they just haven't been included in annotations because they are very rare. The fact that even the moderately deep HEK293 datasets presented here contain evidence for many "novel" junctions, underlines this point. These junctions have presumably been detected in countless prior experiments.

Minor points:

1. I find this statement strange: "In theory, one could increase the abundance of transcripts subject to translation-dependent decay in RNA-Seq libraries by globally inhibiting translation prior to cell lysis." This is not theoretical and has been demonstrated many times, including in the cycloheximide treatment data the authors used here.

2. In their response to reviewer 1, the authors state that the website for GeneSplicer is non-functional, but I had no trouble downloading the source code.

3. Figure 1A -- I find the "projected relative abundance per library" very confusing. What is the basis for the projection of the relative abundance of pioneer and steady state translation RNPs with and without harringtonine?

**Authors’ response**

Major changes:

- We recalculated the normalized RPMs with the equations now included in the Methods section (under “Splice site usage calculations”).
- Using the recalculated RPMs, we have updated the following figures:  Figures 2D-E, Figures 3B-E, Figures 4A-B, Figures 5B-C, Supplemental Figures 4A-C, Figures 6A-B.
- All changes to the text between the original submission and the first revision are highlighted in purple.  New changes between the first and second revisions are highlighted in pink.

Our specific responses to Reviewer #2 comments:

**Reviewer #2**: The revised manuscript by Moore and colleagues is improved in many respects. With some exceptions listed below, the conclusions drawn are generally better justified by the data provided. Despite the improvement, several important issues persist. Please see below for specific comments:

1. I share reviewer #3's concern about the sequencing depth of the RIPiT libraries (6-10 million aligned read pairs per replicate). From Figure 3D and Supplemental Tables 2 and 3, it appears that the majority of the unannotated junctions are represented by only a handful of reads. It's not clear how the reads per million calculations were performed, but it is strange that many junctions have identical RPM counts, with each group of identical RPM counts differing by exactly 0.02558 (see the the stair-step pattern of the EJC curves on the CDF plot in Figure 3D and Supplemental Tables 2 and 3). This suggests that they are not simply the mean of the RPMs from the individual biological replicates, which would be the standard way to represent the data.

This comment prompted us to conduct a comprehensive review of our RPM calculations (both the per-replicate and the averaged per-library RPMs). As predicted by this very astute reviewer, this reexamination revealed an inconsistency between the calculations of EJC and RNA-Seq RPMs. Correction of this inconsistency triggered numerous minor changes in both the text and figures to update the numbers (Figures 2D-E, Figures 3B-E, Figures 4A-B, Figures 5B-C, Supplemental Figures 4A-C, Figures 6A-B).  Of particular importance, Figure 3D no longer exhibits the step pattern.  We note, however, that none of the changes have substantially altered our findings.

Are the RPMs listed for EJC and total RNAseq calculated in the same way (the RNAseq data do not show this pattern of repeated RPM values), and how exactly were they calculated?

The equations used to calculate EJC and RNA-Seq RPM values are now provided in the Methods section under “Splice site usage calculations.”

This all raises the further question of how many of the listed "novel" junctions were represented in multiple RIPiT libraries. About half of the listed events have a RPM of less than 0.25, which across 20 million mapped reads in three libraries (depending on how the RPM calculations were done; based on the step-wise nature of the RPMs, I'm guessing an aggregate of all reads across the three replicates was used as the denominator), would only mean 5 reads per junction in total.

We report 3,275 unannotated junctions in Supplemental Table 3, of which 2,595 junctions are represented in all three of the EJC libraries. Approximately 30% of these junctions had a mean ≥ 5 reads across the EJC replicates.

Also, the response to my previous query says that "read counts in every biological replicate" are provided in Supplemental Table 2 and 3, but there is only one RPM column per condition listed. Junction mapping read counts (along with normalized RPMs) should be provided for all replicates.

We apologize for this previous omission.  The raw junction read counts and per-replicate normalized RPM are now included in both Supplemental Tables 2 and 3.

2. The discussion of the comparison between the novel junctions identified in this study and annotations has improved, but it still conflates detection of junctions with annotation of junctions. The first sentence of the section "EJC libraries capture new exon junctions" says, "We next wondered whether EJC libraries might contain new transcript isoforms that had previously eluded detection due to their low abundance in RNA-seq," but this remains an incorrect representation of the relationship between their data and the annotation compendia used. As previously stated, it is highly likely that most or all of these junctions have been "detected," they just haven't been included in annotations because they are very rare.

We have now changed the *title* and first sentence of this section to read as follows:

*EJC libraries capture previously unannotated exon junctions*

We next wondered whether EJC libraries might contain transcript isoforms that had heretofore escaped annotation due to their low abundance in RNA-Seq.

The fact that even the moderately deep HEK293 datasets presented here contain evidence for many "novel" junctions, underlines this point. These junctions have presumably been detected in countless prior experiments.

We have now eliminated all instances of the word “novel” in the manuscript.  Instead we use the adjective “unannotated” to describe previously unannotated exon junctions.

Minor points:

1. I find this statement strange: "In theory, one could increase the abundance of transcripts subject to translation-dependent decay in RNA-Seq libraries by globally inhibiting translation prior to cell lysis." This is not theoretical and has been demonstrated many times, including in the cycloheximide treatment data the authors used here.

Good point.  We have now changed this sentence to say: “One can increase the abundance of transcripts subject to translation-dependent decay in RNA-Seq libraries by globally inhibiting translation prior to cell lysis [64-65].”  The references provided back up this statement.

2. In their response to reviewer 1, the authors state that the website for GeneSplicer is non-functional, but I had no trouble downloading the source code.

What we previously observed was that the GeneSplicer web interface (https://www.cbcb.umd.edu/software/GeneSplicer/gene_spl.shtml) was non-functional for human sequences, and this is still the case.  And yes, like the reviewer, we were also able to download the source code.  However, the available files are not executable on Mac OSX.  Alternatively, a plug-in for GeneSplicer is available for Ensembl’s Variant Effect Predictor.  Problems installing VEP and executing this plug-in, however, stymied us from further exploring this option. Given the difficulties we encountered and our previous comments on available splice site strength algorithms in the first round of reviews, we have chosen not to pursue this avenue further.

3. Figure 1A -- I find the "projected relative abundance per library" very confusing. What is the basis for the projection of the relative abundance of pioneer and steady state translation RNPs with and without harringtonine?

This graphic serves two functions: (1) It introduces the color schemes for the EJC and RNA-Seq libraries in subsequent panels, and (2) it is meant to illustrate the general trends seen in these later panels.  But we agree that the previous wording may have been confusing as it could potentially be misinterpreted to indicate that there were some actual numbers and calculations underpinning the heights of the bars shown.  So we have now changed the wording to “Hypothetical abundance per library”.

**3^rd^ round**

**Reviewer 2**

The authors have addressed my remaining comments
